# Absence of asymptomatic malaria in a cohort of 133 individuals in a malaria endemic area of Assam, India

**DOI:** 10.1186/s12889-015-2294-0

**Published:** 2015-09-18

**Authors:** Sunil Dhiman, Diganta Goswami, Bipul Rabha, Kavita Yadav, Pronobesh Chattopadhyay, Vijay Veer

**Affiliations:** Department of Medical Entomology, Defence Research Laboratory, Tezpur, Assam India 784 001

**Keywords:** Asymptomatic malaria, Dried blood spots, Rapid diagnostic test (RDT), PCR assay, *Anopheles* vector, VectorTest™

## Abstract

**Background:**

Malaria in northeast India affects children and adults annually. The number of malaria cases reported has declined over the past few years. Nevertheless, it is not clear whether there is an actual decline in parasitaemia or whether asymptomatic malaria infections are on the rise, especially in forested and forest-fringed areas. Asymptomatic malaria forms a parasite reservoir that acts as an epicentre for malaria spread during high-transmission season. Therefore it is important to understand the quantum of asymptomatic malaria infections among the vulnerable population.

**Method:**

Four forest fringed historically malaria endemic villages were selected for the study. A total of 133 individuals without a fever history in the past four weeks were tested for malaria parasite using rapid diagnostic test (RDT), microscopy and polymerase chain reaction (PCR) assay during January – February 2014. Indoor resting *Anopheles* vectors were collected, identified and tested for sporozoite using VectorTest™ panel assay during October 2013 to March 2014, which is a low transmission season for malaria. Social and demographic data were recorded during the study.

**Results:**

Mean age (±SEM) of the participants was 16.1 ± 1.2 years (95 % CI: 13.8–18.4). All participants (100 %) reported to use mosquito nets. Altogether, 43.6 % of participants had education below primary level and only 9 % reported a travel history during the past four weeks. All RDT, microscopy and PCR assays were found negative indicating no asymptomatic malaria parasitaemia. Seven known malaria vector species namely, *Anopheles nivipes*, *An. minimus, An. annularis, An. vagus, An. aconitus, An. philippinensis* and *An. culicifacies,* were recorded in the present study. VectorTest™ sporozoite panel assay conducted on 45 pools (*N* = 224) of vector mosquitoes were found negative for *Plasmodium* sporozoite.

**Discussion:**

Northeastern states of India report asymptomatic malaria parasitemia along with high malaria transmission. *An. minimus* and *An. dirus* are recognised as efficient vectors, but *An. culicifacies*, *An. philippinensis* and *An. annularis* also play role in malaria transmission. Currently all participants were found negative for asymptomatic malaria, however the small sample size may restrict the scope of present results to the population living in more remote areas.

**Conclusion:**

No cases of asymptomatic malaria infections parasitaemia was found in the present study conducted during a low transmission season indicating that asymptomatic malaria parasitaemia may not be prevalent in the region. Mosquito specimens were tested negative for the malaria sporozoites. Study findings encourage the ongoing malaria intervention efforts and recommends similar investigations in different ecological areas involving large populations.

## Background

World Health Organisation (WHO) has estimated that approximately 3.3 billion people are at risk of malaria worldwide, while more than one malaria case occurs per 1000 population in high-risk areas. An estimated 198 million malaria cases (uncertainty range: 124–283 million) and about 0.58 million deaths (uncertainty range: 0.37–0.76) were reported in malaria endemic countries in 2013 [1].

In areas of seasonal malaria transmission, malaria cases start up-surging during the premonsoon season (March-May) and outbreaks occur after the beginning of rainy season (June-August). However during winter season (December-February), the number of malaria infections diagnosed decline [2–4]. Despite low transmission rate, long-term asymptomatic carriers of malaria parasite with very low parasitaemia can be found in the population throughout the winter season [2–4]. In areas of high malaria transmission, regular infection of *Plasmodium* parasite leads to partial immunity and creates asymptomatic carriers [2, 5]. A long term malaria parasite carriage in human population can be critical because the infected individuals may constitute a parasite reservoir in the absence of transmission. Asymptomatic *Plasmodium* species infections are commonly reported in malaria endemic regions of the world including India [6, 7], and the studies have confirmed that asymptomatic parasitaemia sometime in the form of gametocytes occurs in the absence of intense transmission and may persist from one season to another season uninterruptedly [8, 9]. *Plasmodium* gametocytes are formed in the human host but not responsible for clinical symptoms of malaria. However they ensure the transmission of malaria to another human host through mosquito vectors. Asymptomatic carriers do not seek treatment for their infection due to the absence of malaria symptoms, but the malaria parasite remains available for transmission by *Anopheles* mosquitoes once the mosquito density expands during the favourable season [3, 10–12].

Asymptomatic malaria in the endemic areas throughout Asia and Africa is a known phenomenon with year round continuous transmission [4, 13–17]. Children living in endemic settings are frequently exposed to *Plasmodium* species and gradually acquire immunity against malaria. Hence there is comparatively less severe presentation of clinical disease with increased age [16]. Asymptomatic malaria infections pose a great diagnostic challenge as they appear without symptoms. Furthermore, the asymptomatic malaria patients constitute a reservoir of parasites from which the disease may flare during the favourable season [18, 19]. Therefore identification and treatment of individuals with asymptomatic *P. falciparum* in active malaria survey as part of a surveillance intervention strategy could be useful in reducing the parasite reservoir and will have an impact on disease transmission during the peak malaria season [3, 6, 7, 20].

Asymptomatic malaria parasitaemia in endemic areas of India is geographically distributed but remains entrenched among low socioeconomic status population groups living in forest and rural areas [3, 4, 6, 17, 18]. A recent study performed in eastern India using PCR assay on 1,040 inviduals displayed that a significant proportion (8.4 %) of the sample was infected with *P. falciparum* without any clinical manifestations [6]. In another study in Odisha, 16.5 % (12,045) of the study population was positive for malaria parasite and *P. falciparum* accounted for 89.1 % of the total positives. Gametocytes were detected in 7.7 % of the positive cases. The study highlighted that 78.9 % of the parasite carriers were afebrile and without any other symptom [7].

Malaria transmission is perennial in the villages of Assam-Arunachal Pradesh interstate border and symptomatic as well as asymptomatic malaria infections are detected throughout the year with high slide positivity rate mainly due to *P. falciparum* [4, 11, 18]. Both symptomatic and asymptomatic malaria is reported but asymptomatic malaria carriers were found responsible for persistent malaria transmission in the study region [4, 15]. A recent study conducted in the study area revealed that slide positivity rate (SPR) for symptomatic and asymptomatic malaria was 26.1 % and 5.0 % respectively. The study further revealed that *P. falciparum* was the major asymptomatic malaria parasite and SPR was indifferent among the different seasons. Monthly parasitic index remains highest during July (20.3) and least during the month of January (1.9), whereas SPR was highest during June-July and lowest during the months of March and April. The study recorded a high slide *falciparum* rate (SfR) of 20.8 in the study area [4]. In India, the reported malaria related morbidity and mortality has decreased in the recent years, but it is important to ascertain the existence of asymptomatic malaria infections, especially in forested and forest fringed areas.

In the study area, *Anopheles minimus* and *An. fluviatilis* have been found to be associated with perennial malaria infection, while other vectors such as *An. annularis*, *An. culicifacies* and *An. philippinensis* play secondary roles during the peak malaria season [4, 10, 11]. The vector prevalence and their sporozoite positive assay were performed to understand known malaria vector density and whether or not these are involved in malaria transmission during the low transmission season. The primary objective was to study the prevalence of asymptomatic parasitaemia and the secondary objective was to study the presence of known malaria vectors and their possible role in malaria transmission in four selected forest-fringed villages of a malaria endemic area near Assam-Arunachal border in northeast India during low transmission season.

## Methods

### Selection of the study area

**Fig. 1:**
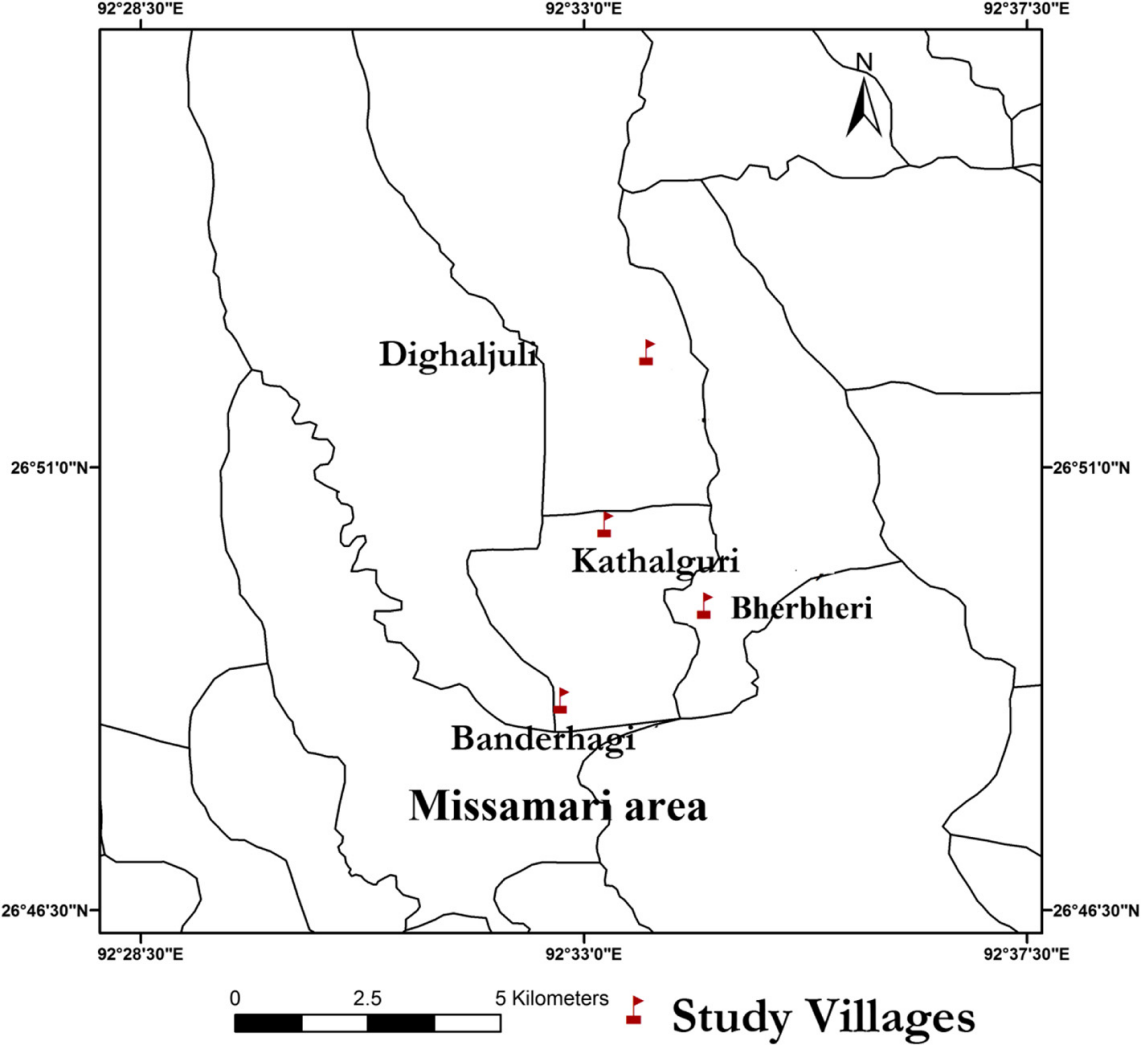
GPS map depicting the study villages in Missamari area in Assam, India

This study was conducted in four neighbouring malaria endemic villages (Banderhagi, Bherbheri, Kathalguri and Dighaljuli) situated in the forest-fringed Missamari area, Assam (26°48'00.0"N to 92°36'00.0"E) having previous record of annual malaria transmission [4, 11]. The study villages are situated near Assam-Arunachal Pradesh interstate border, have a population of about 1200 persons and inhabited mainly by tribals (Fig. 1). High malaria incidence is recorded from March to September every year and human transmission is facilitated mainly by *An. dirus, An. minimus, An. annularis, An. philippinensis* and *An. culicifacies* mosquitoes [4, 11, 21–25]. For the current study, a door-to-door survey was conducted and included 133 individuals of different age groups. Only those individuals that had not been ill with fever or confirmed malaria within the past four weeks were included in the study. Further those who did not agree to give a sample were not included in the study. Informed consent by finger impression or signature was obtained from the study participants or their guardian. The study was approved by the institutional human ethical committee of LGNBRIMH, Tezpur.

### Blood sample collection

Human blood samples were collected via convenience sampling during January - February 2014 and tested for malaria using immunochromatography rapid diagnostic test kits (OptiMAL-IT, Diamed AG, Switzerland) at the spot according to the manufacturer’s instructions, while both thick and thin smears of blood were also taken on glass slides for microscopic examination. Minimum sample size for present study was determined by assuming a baseline prevalence of 8.4 % [6] and sensitivity of test as 97 % in a population of 1200 individuals at 95 % confidence interval. The blood smears taken on glass slides were stained using Giemsa stain and observed under microscope for the presence of malaria parasite species by at least two independent technicians. During the blood sample collection, a few drops (2–4) were taken on FTA classic cards (Whatman, Sweden) and air-dried. The FTA cards with the dried blood spots were stored in an airtight plastic packet and later kept in −20 °C in freezer until DNA extraction for PCR assay. RDTs were used to detect the malaria parasite in the field itself and subsequently confirmed by using microscopy and PCR in laboratory. The performance of RDT depends upon malaria parasitaemia and it may yield a false negative result in asymptomatic cases with low malaria parasitaemia. Similarly, microscopic examination could also miss low levels of malaria parasitaemia even to expert examiners. The PCR method has been found to be very sensitive and specific to detect low malaria parasitaemia at levels undetectable by microscopy. Therefore to completely eliminate the chance of any false result, the blood from all subjects was analyzed using an RDT, microscopy and PCR assay. A simple questionnaire was filled for each participant recording the age, gender, any travel within the past four weeks, education level and use of anti-mosquito measures such as coil, repellents, etc. The bed net usage was ascertained by asking the question, “Do you sleep inside the bed net?”

### *Anopheles* mosquito collection and malaria sporozoite panel assay

*Anopheles* mosquitoes were collected from human dwellings using hand catch collection of individual adult mosquito resting inside the houses with the help of an aspirator tube and flashlights. Mosquito collection was made on a monthly basis from October 2013 to March 2014 for thirty minutes each in two randomly selected index houses in each study village with the density expressed as per man hour resting density (PMHRD) for each species. Collected female known vectors *Anopheles* were identified using standard keys and stored in labelled eppendorf tubes (0.5 ml) singly for sporozoite panel assay using VectorTest™ (Vector Test System, Inc., USA; Lot MALK050113) malaria sporozoite panel assay. For the assay, at least 2–5 female mosquitoes of same species were put in the conical plastic tube (provided with the test kit) and 250 μl grinding solution was added as per the manufacturer’s instructions. The content was ground and homogenised with plastic pestle. Panel assay strip was put into the mosquito suspension and reading was recorded as per standard instruction. Currently, a total of 224 mosquitoes comprising of *An. nivipes* (*n* = 38), *An. minimus* (*n* = 5), *An. annularis* (*n* = 46), *An. vagus* (*n* = 105), *An. aconitus* (*n* = 11), *An. philippinensis* (*n* = 17) and *An. culicifacies* (*n* = 2) were tested for the presence of *Plasmodium* sporozoite. Previous studies conducted to evaluate the performance of VectorTest™ panel assay kit have showed that the VectorTest™ panel assay was highly sensitive and specific, and proved technically simpler to perform and interpret than the ELISA based method [26, 27]. Studies have proved that VectorTest™ panel assay showed 92 % sensitivity, 98.1 % specificity and 97.8 % overall accuracy under test conditions and thereby performed to an acceptable level of reliability and has been recommended for use in field in rapid survey of malaria vectors [27].

### DNA extraction and PCR assay

DNA extraction from the blood samples collected on FTA cards was done using Qiagen kit (Qiagen, Hilden, Germany) following the standard manufacturer procedure. PCR for *Plasmodium* 18 s rRNA smaller sub-unit was performed using *Plasmodium* genus-specific primers PLU5/PLU6 as described previously [24, 28, 29], whereas the reaction conditions for PCR were similar to as standardised in our recent studies [24, 30]. DNA extracted from the known *P. falciparum* samples was used as positive control while from confirmed malaria negative sample was used as negative control for PCR assays. PCR products were analysed in 2 % agarose gel electrophoresis (Sigma Aldrich, USA) and visualised using UV transilluminator (Syngene G-box, USA).

### Statistical analysis

The average age of participants was determined by calculating the mean and expressed as mean ± standard deviation (mean ± SD). Known *Anopheles* vector density was expressed in mean ± standard error mean (mean ± SEM) and presented as PMHRD. The PMHRD of mosquito vectors during the study months was compared using ANOVA. All statistical analysis was performed using GraphPad Prism version 6.00 (GraphPad Software, La Jolla California USA, www.graphpad.com).

## Results

### Study population demography and malaria diagnosis

Demographic profile of the study population (*N* = 133) has been shown in Table 1. The average age of the study participants was 16.1 ± 10.6 years (95 % CI- 14.3–17.9), of which 57 (42.9 %) were females. All the participants (100 %) reported to use mosquito nets while 77.3 % used anti-mosquito measures such as repellents coils, creams, spray etc. Altogether 57.1 % had education above primary level and only 9 % reported a travel history during the past four weeks. All the RDT’s were found negative for malaria diagnosis in the present study. The microscopic examination also revealed that none of the participants had *Plasmodium* infection. Similarly all PCR assays for *Plasmodium* detection in the study participants were negative.

**Table 1:** Demographic profile of study population (*N* = 133)

Variable	Outcome
Age (mean ± SD; 95 % CI)	16.1 ± 10.6 (Range: 1–60; 14.3–17.9)
Sex (male/female)	76/57
Mosquito net using (%)	133 (100)
Education	
No education (%)	7 (5.3)
Some primary education (%)	14 (10.5)
Primary (%)	37 (27.8)
Above primary (%)	75 (57.1)
Travel outside area within past 4 weeks (%)	12 (9.0)
Using anti-mosquito measures such as coils, repellents, cream etc. (%)	103 (77.4)

*N* number, *SD* standard deviation, *CI* confidence interval, % percentage

### *Anopheles* mosquito density and sporozoite detection assay

Known *Anopheles* vector mosquitoes species PMHRD recorded in the current study have been shown in Table 2. A total of seven species namely, *An. nivipes*, *An. minimus, An. annularis, An. vagus, An. aconitus, An. philippinensis* and *An. culicifacies* were recorded from October 2013 to March 2014. No *Anopheles* vector species was recorded in the months of January and February 2014. *An. vagus* was predominant species during October 2013 (PMHRD =15.5 ± 3.5; *p* = 0.0004) and March 2014 (PMHRD =5.8 ± 2.5; *p* = 0.04) and its PMHRD was found to be statistically higher than the other *Anopheles* species (Table 2). On the other hand prominent malaria vectors *An. minimus* and *An. culicifacies* were least abundant and recorded during the month of October (PMHRD =1.3 ± 0.6) and March only (PMHRD =0.5 ± 0.3). During the remaining months PMHRD of *Anopheles* species did not differ significantly. The VectorTest™ sporozoite panel assay conducted on 45 pools (*N* = 224) comprised of recorded seven known vector species for the presence of malaria parasite sporozoite did not detect any pool positive for malaria parasite.

**Table 2:** Known *Anopheles* vector per man hour resting density (PMHRD) recorded during the study period (Oct 2013- Mar 2014)

Months		*Anopheles* vectors							F (p)/R^2^
	Variables	*An. nivipes*	*An. minimus*	*An. annularis*	*An. vagus*	*An. aconitus*	*An. philippinensis*	*An. culicifacies*	
Oct 2013	Mean ± SEM	9.5 ± 1.7	1.3 ± 0.6	6.8 ± 1.8	15.5 ± 3.5	0.8 ± 0.5	NR	NR	10.0 (0.0004)/0.7
	COV (%)	36.0	100.7	53.2	44.9	127.7			
	95 % CI	4.1–15.0	–0.8–3.3	1.0–12.5	4.5–26.6	−0.8–2.3			
Nov 2013	Mean ± SEM	NR	NR	3.0 ± 0.8	3.0 ± 0.9	1.3 ± 0.5	1.3 ± 0.8	NR	2.1 (0.2)/0.3
	COV (%)			54.4	60.9	81.7	120		
	95 % CI			0.4–5.6	0.1–5.9	−0.7–3.0	−1.1–3.6		
Dec 2013	Mean ± SEM	NR	NR	1.5 ± 0.6	2.0 ± 0.4	1.0 ± 0.4	0.8 ± 0.5	NR	1.3 (0.3)/0.2
	COV (%)			86.1	40.8	81.7	127.7		
	95 % CI			−0.6–3.6	0.7–3.3	−0.3–2.3	−0.8–2.3		
Jan 2014	No known malaria vector recorded								
Feb 2014	No known malaria vector recorded								
Mar 2014	Mean ± SEM	NR	NR	0.3 ± 0.3	5.8 ± 2.5	NR	2.3 ± 0.7	0.5 ± 0.3	3.9 (0.04)/0.5
	COV (%)			200	86.8		42.6	115.5	
	95 % CI			−0.5–1.0	−2.2–13.7		0.7–3.8	−0.4–1.4	

*SEM* standard error mean, *NR* not recorded, *COV* coefficient of variance, *CI* confidence interval

## Discussion

Present results showed that asymptomatic malaria infections are absent in this sample, which contrasts with previous reports and indicates that all positive cases detected during passive surveillance are true clinical malaria cases [31, 32]. Asymptomatic malaria parasitaemia is generally found in adults due to a certain level of immunity which develops with age and frequent exposure to vector mosquitoes [6].

A previous study conducted in Tanzania reported that asymptomatic malaria was not found in children [9]. It has been observed that asymptomatic *Plasmodium* infection can persist in semi-immune individuals for about two years in the areas where possibility of re-infection is excluded. The persistence of asymptomatic *P. falciparum* during the period of non-transmission or inter-seasonally have been reported in the studies conducted in many Asian countries [6, 7].

PCR assay has been found highly useful in detecting malaria cases as compared to microscopy and RDT [30]. The sensitivity and specificity of PCR assay were 97 % and 100 %, respectively, whereas positive predictive value, negative predictive value and false discovery rate were 100 %, 90 % and 0 %, respectively [30]. The RDT is likely to miss some asymptomatic cases as the threshold levels of parasitaemia in asymptomatic infection may be up to <100 parasite/μl, but the cases missed by RDT are expected to be identified by PCR assay [9, 29, 30]. There was consistency in the results obtained in microscopy, RDT and PCR, which suggests that the results achieved in the present study are valid.

The previous studies conducted on mosquito vectors in the study area suggested that many efficient *Anopheles* vectors operate throughout the year and support perennial transmission of malaria. Malaria vectors *An. dirus*, *An. minimus* and *An. annularis* are well established in the region, occur most of the year and contribute a major share in malaria transmission [11, 23, 24, 33–36]. In the present study previously reported malaria vectors *An. philippinensis*, *An. minimus*, *An. vagus*, *An. culicifacies* and *An. annularis* were found to form majority of the total anopheline collection. Although none of the mosquito specimens were found positive for malaria sporozoite but considerable vector density was recorded even in the months of low malaria transmission. *An. vagus*, though not a well reputed malaria vector, was recorded in high density during the months of October and March. Unlike other studies, *An. dirus* was not recorded, whereas *An. minimus* and *An. culicifacies* were sparse in prevalence and recorded in the month of October and March respectively. More recent studies conducted in the northeast region of India have also suggested that *An. annularis* and some other *Anopheles* species of little malaria importance are involved in malaria transmission and their high density throughout the year may establish them as important vectors later [21, 24, 35]. Present results suggested that there was no indigenous malaria transmission due to the absence of malaria parasite pool among the study population. Although the present vector data was recorded from a small subset of the region during winter months, hence cannot be generalised for other areas of the northeast region.

During the recent years, there has been remarkable scale-up of malaria control intervention particularly in the endemic countries. Extraordinary growth in the number of individuals using insecticide-treated bed nets, houses allowing indoor residual spraying (IRS) and improvement in the health infrastructure has resulted in a significant decline in malaria related mortality and morbidity between 2000 and 2014 globally [1]. National Rural Health Mission (NRHM) of India has emphasized the active surveillance of malaria and improvement in case management through the use of effective antimalarials, which probably interrupted malaria transmission and spread from malaria hot spots, and reduced the parasite reservoir harbored by asymptomatic individuals in northeast India [18, 37].

In the current study, the majority of the enrolled participants (53.4 %; *N* = 71) were above 16 years of age which make the findings generalised that asymptomatic parasite is not present in the study area. Adult humans are expected to have much asymptomatic parasitaemia as it develops slowly when the child grows up in high perennial malaria transmission areas [6, 16, 38–40]. The study area has reported high asymptomatic malaria incidence during the previous years [4], but the present study has not found any asymptomatic infection. For the first time, this study has established the absence of asymptomatic malaria and emphasized the success of malaria control programmes undertaken during the recent past in the study area.

None of the mosquito specimens were positive for sporozoite indicating no malaria transmission in the study area during the study period. Efficient and annual malaria vectors such as *An. minimus* and *An. culicifacies* were infrequent and recorded in very low density during the winter season. Furthermore all the three methods (RDT, microscopy and PCR) used to test for malaria among participants were consistent in performance and were consonant to each other. However the small sample size of the population taken from a limited area may restrict the generalizability of present results to the population living in other areas of northeast India. The mosquitoes were collected in indoor resting only, which may have limited the collection of exophagic vectors such as *An. dirus* in the current study.

## Conclusions

The study suggested that asymptomatic malaria infections are not present which in turn encourages the ongoing malaria intervention efforts. Although many efficient vectors were present during the study, none was found involved in malaria transmission thus posing an ‘*anophelism without malaria*’ [41] like situation. The present findings cannot be generalised to the population living in other remote and forest areas of northeast India. More similar studies involving a large population from different ecological areas and testing endophilic as well as exophilic *Anopheles* vectors could augment in arriving at concrete and generalised conclusion.
